# Supplementary material for: Characterization of Medusavirus encoded histones reveals nucleosome-like structures and a unique linker histone
Source: Nat Commun. 2024 Oct 23;15:9138. doi: 10.1038/s41467-024-53364-5 (PMC11500106; doi:10.1038/s41467-024-53364-5)
Supplement: Supplementary file 1 — Supplementary Information [file 41467_2024_53364_MOESM1_ESM.pdf]

Supplementary Information

Characterization of *Medusavirus* encoded histones reveals nucleosome-like structures and a unique linker histone

Chelsea Marie Toner[1†], Nicole Marie Hoitsma[1,2†], Sashi Weerawarana[1], and Karolin Luger[1,2 *]

[1]Department of Biochemistry; University of Colorado at Boulder, 80309 Boulder, Colorado

[2]Howard Hughes Medical Institute, Chevy Chase, Maryland

† These authors contributed equally to this work

*Author for Correspondence: karolin.luger@colorado.edu

Supplementary Figure 1. Complete sequence alignment and secondary structure prediction of *Medusavirus medusae* histones.

Supplementary Figure 2. MM-NLP preparation for Cryo-EM.

Supplementary Figure 3. Biochemical analysis of *Medusa medusae* octamers and tri-nucleosomes.

Supplementary Figure 4. Cryo-EM analysis of Native and GraFix MM-NLP$_{207\ bp}$.

Supplementary Figure 5. Electrostatic surface representation comparison of eNuc to viral NLPs.

Supplementary Figure 6. Electrostatic surface representation comparison of host *A. castellanii* H1.1 and *X. laevis* H1.0 to *Medusa medusae* linker histone H1.

Supplementary Figure 7. Biochemical analysis of *Mus musculus* and *Medusavirus medusae* histone H1.

Supplementary Table 1. Summary of cryoEM data collection and refinement.

a.

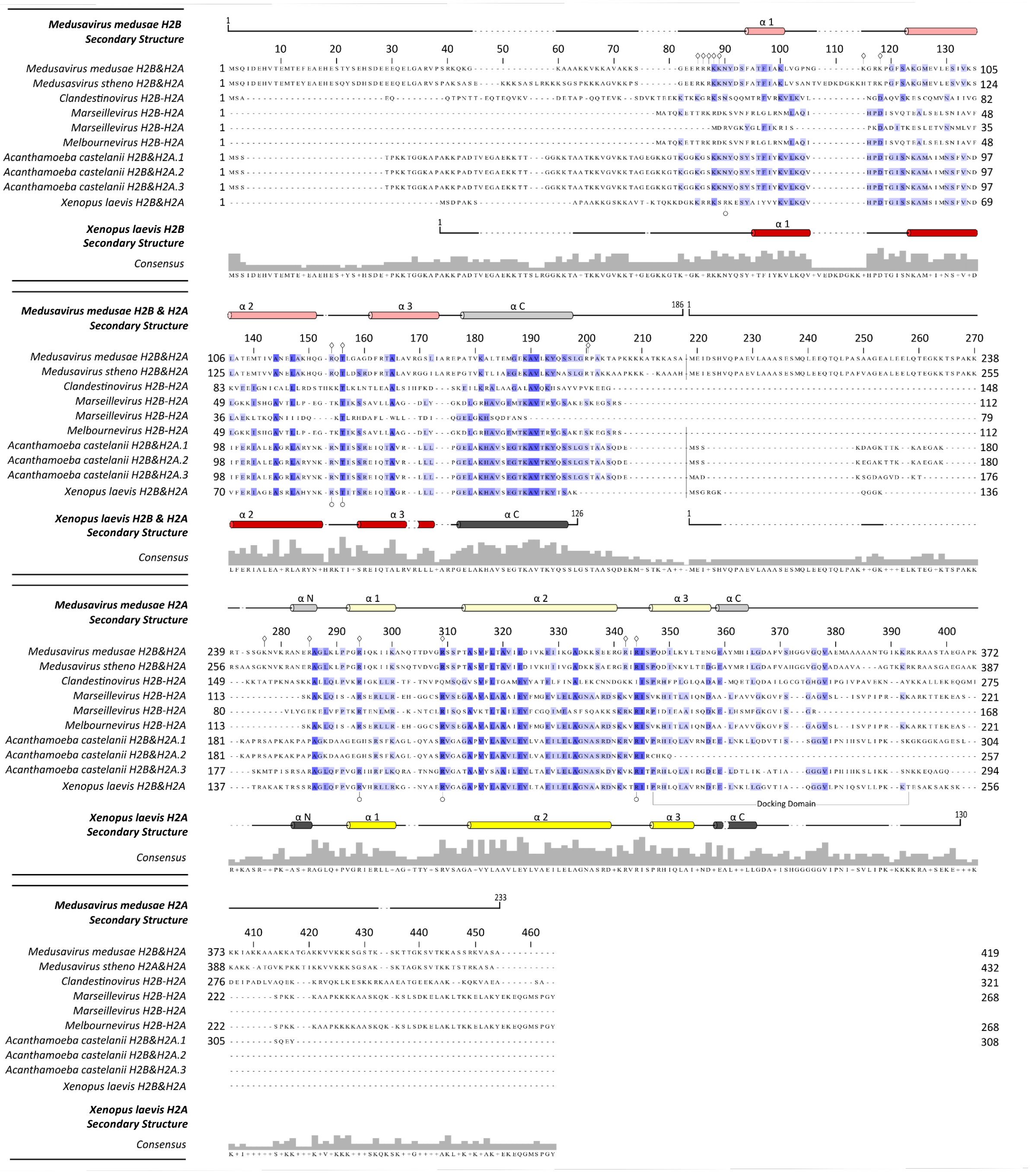

Medusavirus medusae H2B
Secondary Structure
Medusavirus medusae H2B&H2A
Medusavirus stheno H2B&H2A
Clandestinovirus H2B-H2A
Marseillevirus H2B-H2A
Marseillevirus H2B-H2A
Melbournevirus H2B-H2A
Acanthamoeba castelanii H2B&H2A.1
Acanthamoeba castelanii H2B&H2A.2
Acanthamoeba castelanii H2B&H2A.3
Xenopus laevis H2B&H2A
Xenopus laevis H2B
Secondary Structure
Consensus
Medusavirus medusae H2B & H2A
Secondary Structure
Xenopus laevis H2B & H2A
Secondary Structure
Medusavirus medusae H2A
Secondary Structure
Xenopus laevis H2A
Secondary Structure
Docking Domain
α N
α 1
α 2
α 3
α C

b.

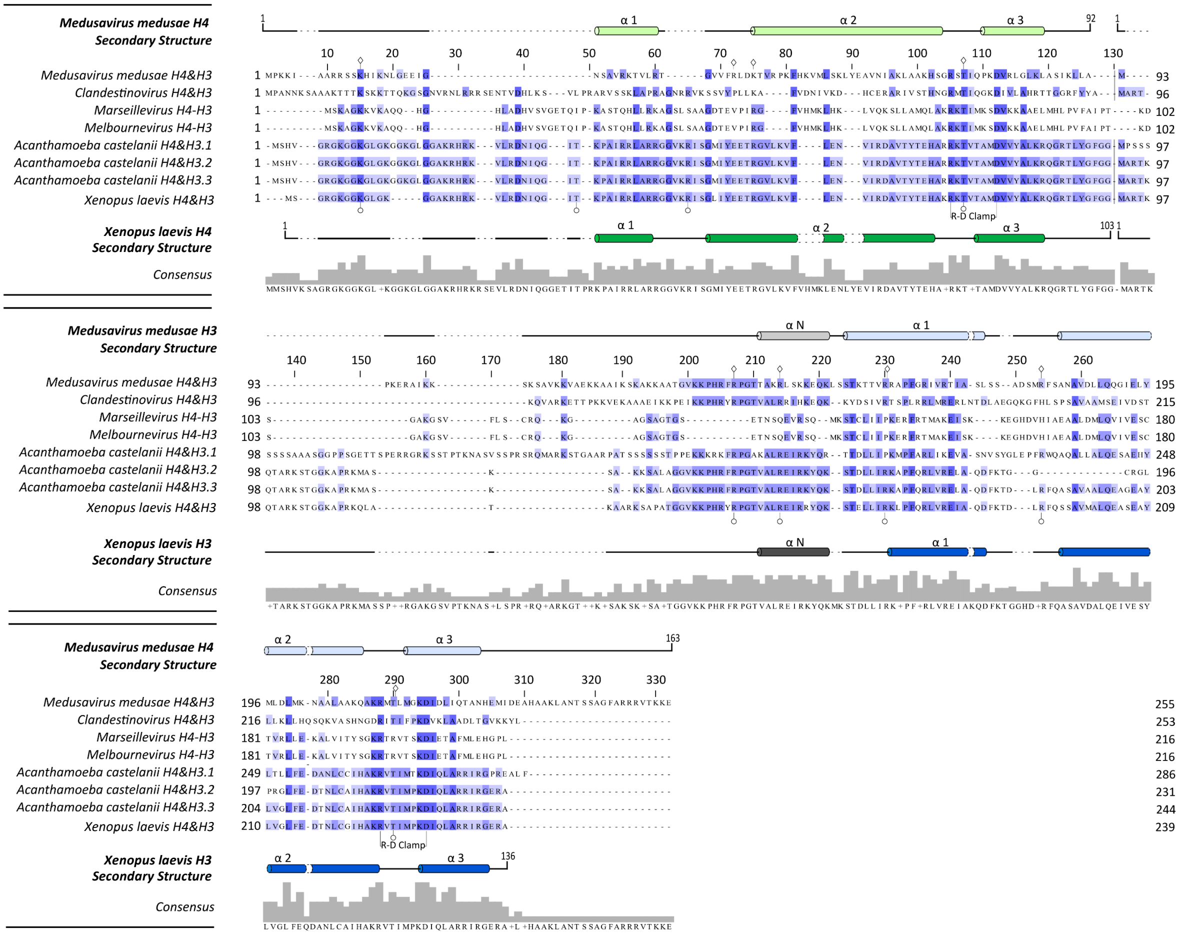
Medusavirus medusae H4
Secondary Structure
α 1
α 2
α 3
Medusavirus medusae H4&H3
Clandestinovirus H4&H3
Marseillevirus H4-H3
Melbournevirus H4-H3
Acanthamoeba castelanii H4&H3.1
Acanthamoeba castelanii H4&H3.2
Acanthamoeba castelanii H4&H3.3
Xenopus laevis H4&H3
R-D Clamp
Xenopus laevis H4
Secondary Structure
Consensus
Medusavirus medusae H3
Secondary Structure
α N
Xenopus laevis H3
Secondary Structure
Medusavirus medusae H4
Secondary Structure

c.

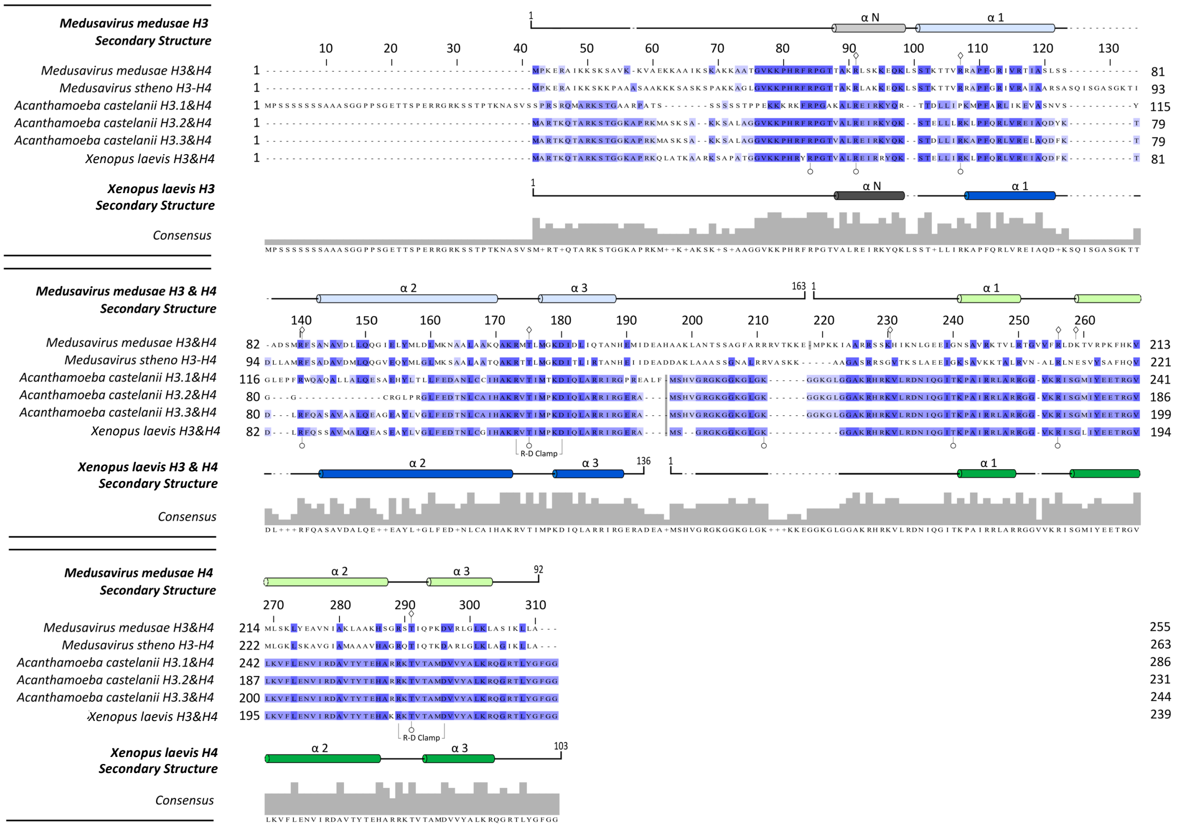
Medusavirus medusae H3
Secondary Structure
α N
α 1
Medusavirus medusae H3&H4
Medusavirus stheno H3-H4
Acanthamoeba castelanii H3.1&H4
Acanthamoeba castelanii H3.2&H4
Acanthamoeba castelanii H3.3&H4
Xenopus laevis H3&H4
Xenopus laevis H3
Secondary Structure
Consensus
Medusavirus medusae H3 & H4
Secondary Structure
α 2
α 3
R-D Clamp
Xenopus laevis H3 & H4
Secondary Structure
Medusavirus medusae H4
Secondary Structure
Xenopus laevis H4
Secondary Structure

d.

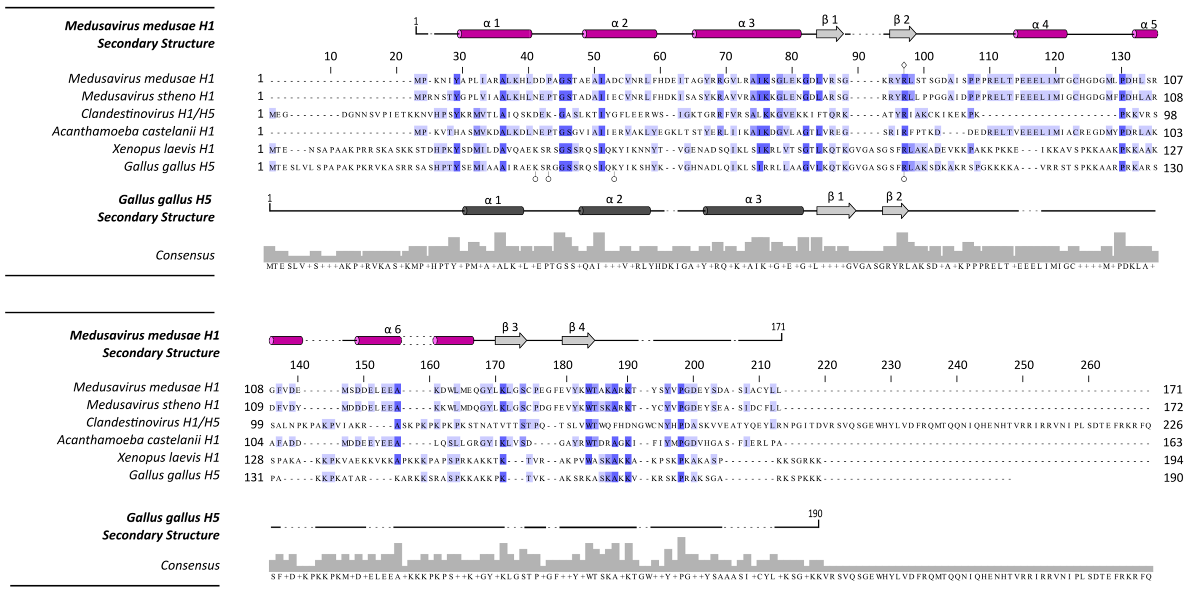

e.

	pI
Medusavirus medusae A0A3T1CWP1	5.0
Medusavirus stheno H1 A0A7S7YFS7	5.3
Clandestinovirus H1/H5 A0A8F8PQR7	10.3
A. castellanii H1.1 L8GT86	4.9
A. castellanii H1.2 L8GK55	10.5
A. castellanii H1.3 L8GITG8	6.4
X. laevis H1.0 Q6NVM0	10.9
G. gallus H5 P02259	12.2

f.

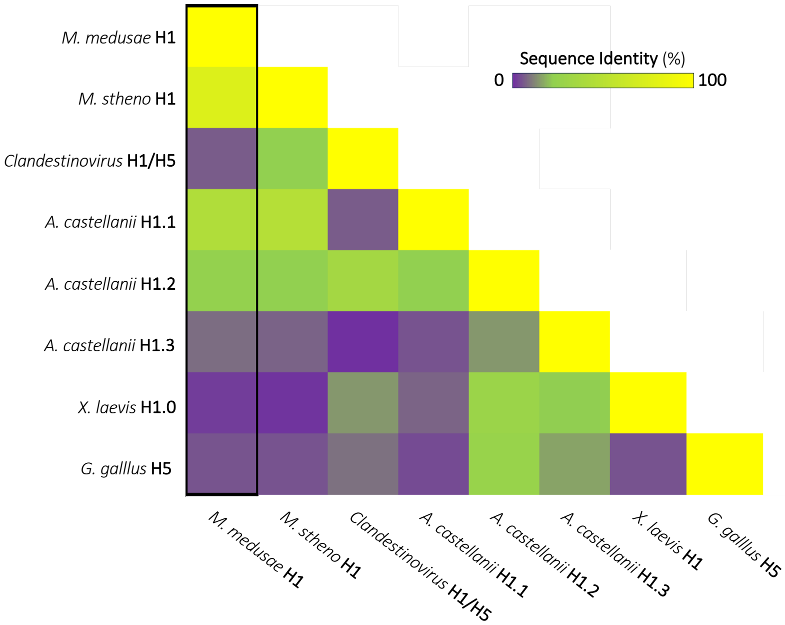

Supplementary Figure 1. Complete sequence alignment and secondary structure prediction of *Medusavirus medusae* histones.

Viral histone dimer pairs (or doublets) H2B-H2A and H4-H3 were aligned against *A. castellanii* and *X. laevis* histones using HHPRED's multiple sequence alignment tool, ClustalΩ. Conservation

of each residue within the alignment is represented by blue shading, where darker blue signifies a greater conservation. Known α helices of *X. laevis* are shown in dark colored tubes (H2B-red, H2A-yellow, H4-green, H3-blue). Predicted α helices of MM (light colored tubes) were generated using HHPRED's Quick 2D prediction web server.

(a) Complete sequence alignment of H2B and H2A viral histones (*Mamonoviridae* and *Marseilleviridae* families) against viral host histones *A. castellanii,* and *X.laevis*.

(b) Complete sequence alignment of H4 and H3 viral histones (excluding *M. stheno* doublet) against viral host *A. castellanii*, and *X, laevis*.

(c) Complete sequence alignment of H3 and H4 viral histones (including *Medusavirus stheno* H3-H4 doublet) against viral host *A. castellanii*, and *X. laevis*. This differs from previous alignment in order of histone pairs (H3-H4 instead of H4-H3).

(d) Viral putative linker histone H1 was aligned against *A. castellanii, X. laevis*, and *Gallus gallus* H5. Known α helices of *X. laevis* H1 are shown in dark grey colored tubes. Predicted α helices of MM (pink) were generated using HHPRED's Quick 2D prediction web server.

(e) Isoelectric point (pI) of each predicted and known linker histone H1.

(f) Heat map comparing percent identity of predicted viral linker histone H1/H5 and eukaryotic H1/H5 sequences. MM-putative H1 is outlined in black.

Related to Figure 1. Source data are provided as a Source Data file.

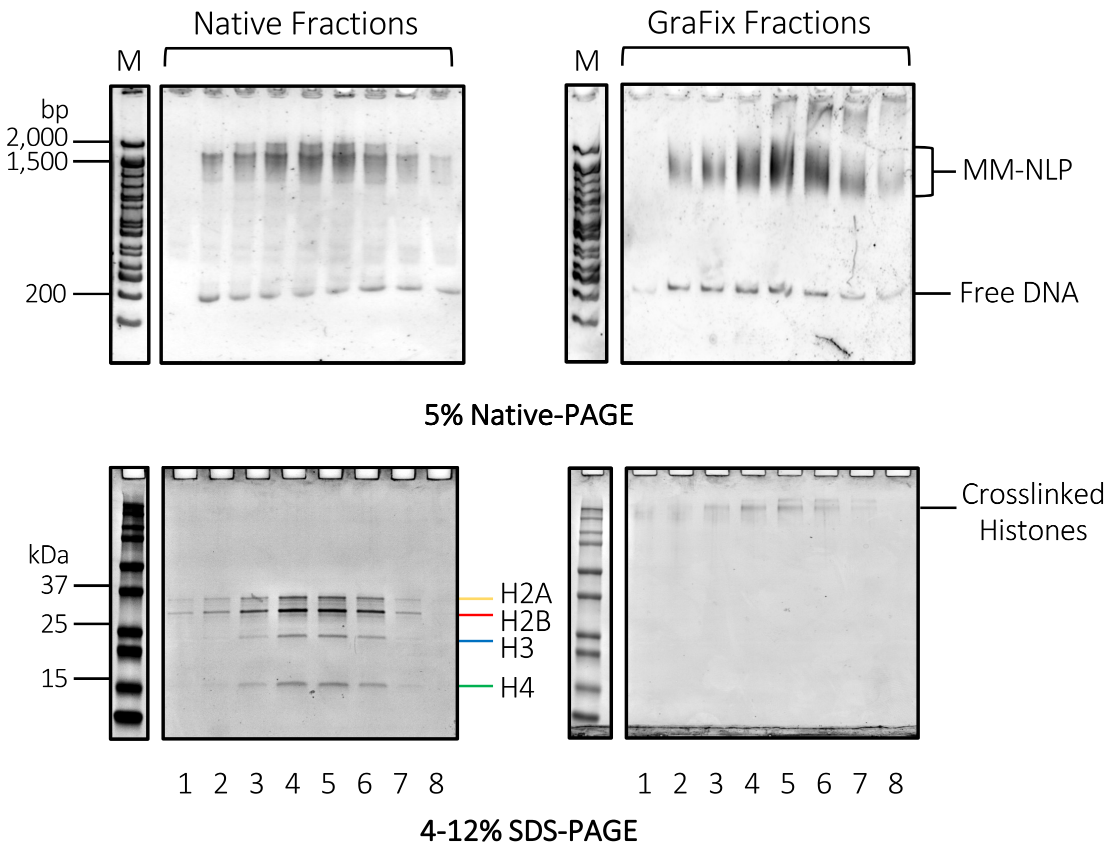

Supplementary Figure 2. MM-NLP preparation for Cryo-EM.

Sucrose gradient sedimentation and GraFix of MM-NLP with 207 bp DNA ($MM\text{-}NLP_{207W}$). Fractions of each were analyzed by 4-12% SDS-PAGE stained with BlazinBlue (protein visualization) and 5% Native-PAGE stained with SYBRGold (DNA visualization) to determine composition of particles. Experiment was repeated independently more than three times with similar results. Related to Figure 2 and 3. Source data are provided as a Source Data file.

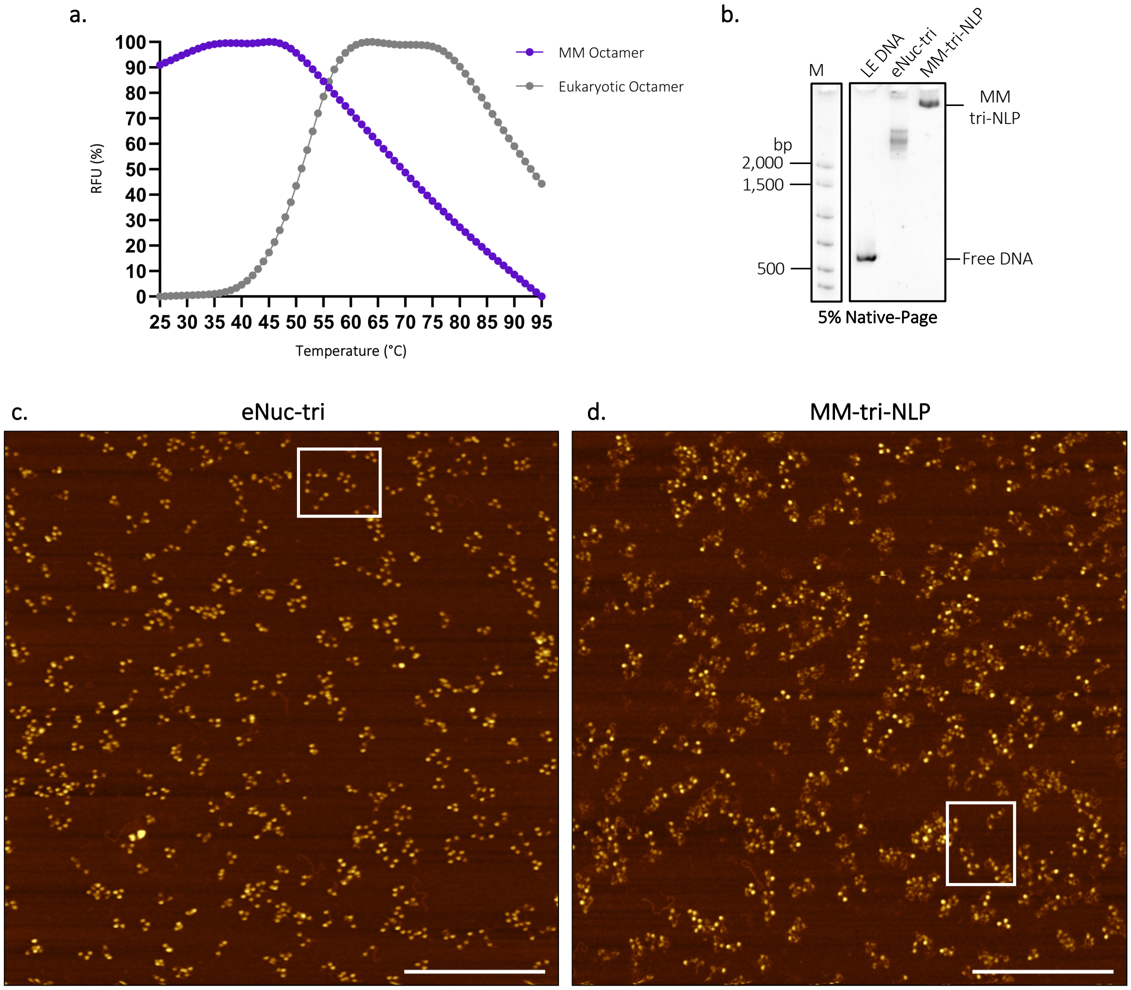

Supplementary Figure 3. Biochemical analysis of *Medusa medusae* octamers and tri-nucleosomes.

(a) Thermal shift stability of MM and eukaryotic octamer utilized in formation of NLP. The raw relative fluorescence units were normalized for plotting (n=1).

(b) MM tri-nucleosomes (MM-tri-NLP) and eukaryotic tri-nucleosomes (eNuc-tri) on LE DNA. Experiment was repeated independently more than three times with similar results.

(c) (d) Representative AFM topography images of (c) eNuc-tri and (d) MM-tri-NLP, white squares represent particles shown in Figure 2G. Scale bar = 500 nm.

Related to Figure 2. Source data are provided as a Source Data file.

a.

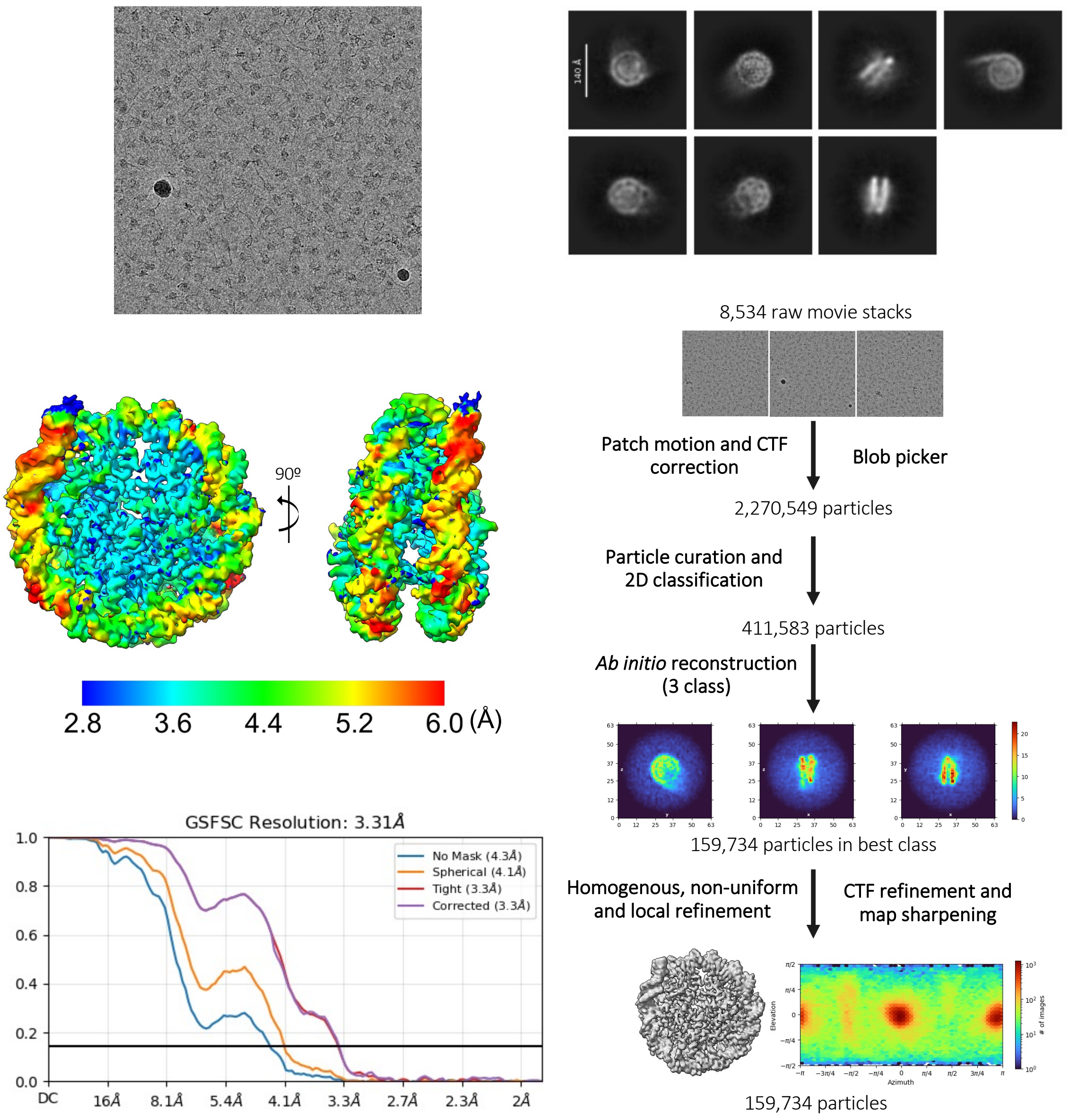

140 Å
8,534 raw movie stacks
Patch motion and CTF correction
Blob picker
2,270,549 particles
Particle curation and 2D classification
411,583 particles
Ab initio reconstruction (3 class)
159,734 particles in best class
Homogenous, non-uniform and local refinement
CTF refinement and map sharpening
159,734 particles
90º
2.8
3.6
4.4
5.2
6.0
(Å)
GSFSC Resolution: 3.31Å
No Mask (4.3Å)
Spherical (4.1Å)
Tight (3.3Å)
Corrected (3.3Å)
DC
16Å
8.1Å
5.4Å
4.1Å
3.3Å
2.7Å
2.3Å
2Å
Elevation
Azimuth
# of images

b.

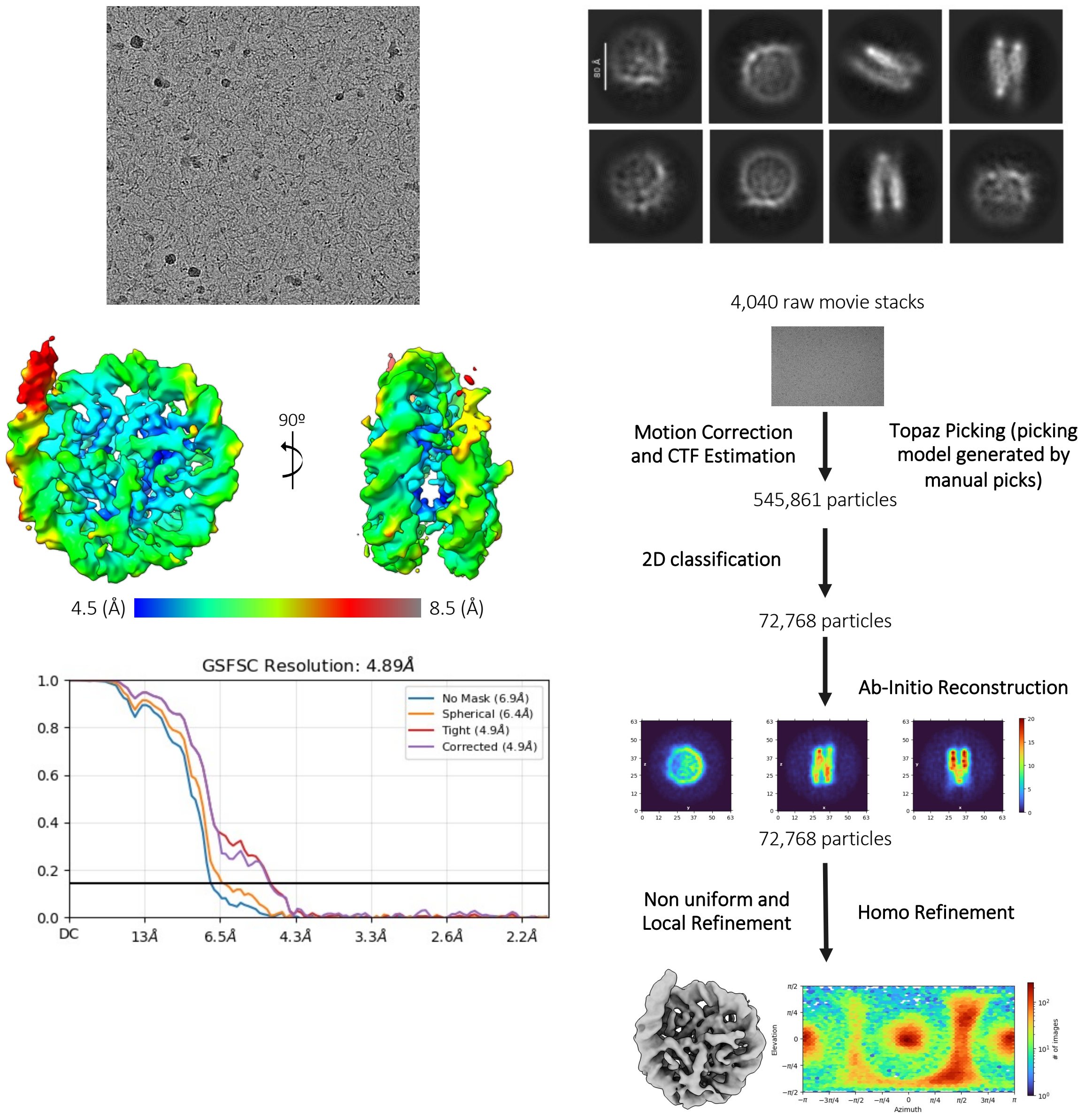

Supplementary Figure 4. Cryo-EM analysis of Native and GraFix MM-NLP$_{207\ bp}$.

(a) Raw micrograph of GraFix MM-NLP$_{207}$, 2D class averages generated from represented dataset (scale bar = 140 Å), 3D structure of MM-NLP with local resolution map, FSC curve, and CryoSPARC data processing flow chart. An FSC cutoff of 0.143 was used to determine resolution. Bump in FSC curve at ~5 Å is due to the flexible free-DNA arm.

(b) Raw micrograph of native MM-NLP$_{207}$, 2D class averages generated from represented dataset (scale bar = 80 Å), 3D structure of MM-NLP with local resolution map, FSC curve, and CryoSPARC data processing flow chart.

Related to Figures 3, 4 and 5.

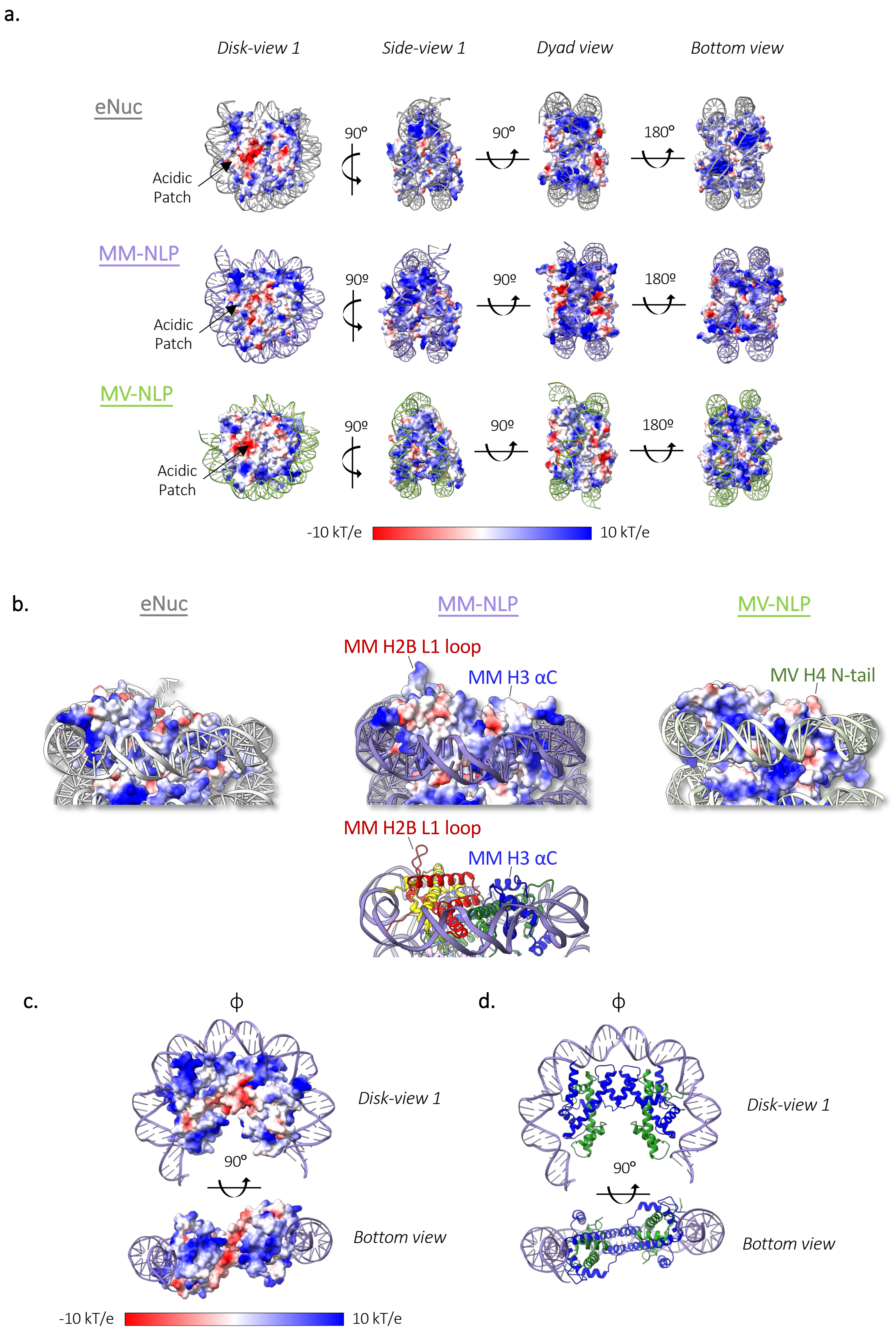
a.
Disk-view 1
Side-view 1
Dyad view
Bottom view
eNuc
Acidic Patch
90°
90°
180°
MM-NLP
Acidic Patch
90º
90º
180º
MV-NLP
Acidic Patch
90º
90º
180º
-10 kT/e
10 kT/e
b.
eNuc
MM-NLP
MV-NLP
MM H2B L1 loop
MM H3 αC
MV H4 N-tail
MM H2B L1 loop
MM H3 αC
c.
ϕ
Disk-view 1
90°
Bottom view
-10 kT/e
10 kT/e
d.
ϕ
Disk-view 1
90°
Bottom view

Supplementary Figure 5. Electrostatic surface representation comparison of eNuc to viral NLPs.

(a) Charged surface representation of histones from the eNuc (PDB ID: 3LZ0), MM-NLP and Melbournevirus-NLP (MV-NLP; PDB ID: 7N8N) in different orientations.

(b) Oblique view of three nucleosomes to highlight contributions of the MM H2B L1 loop and the MM H3 αC helix

(c) Charged surface and (d) cartoon representation of MM $(H3\text{-}H4)_2$ tetramer with 60 bp of DNA.

Related to Figure 5.

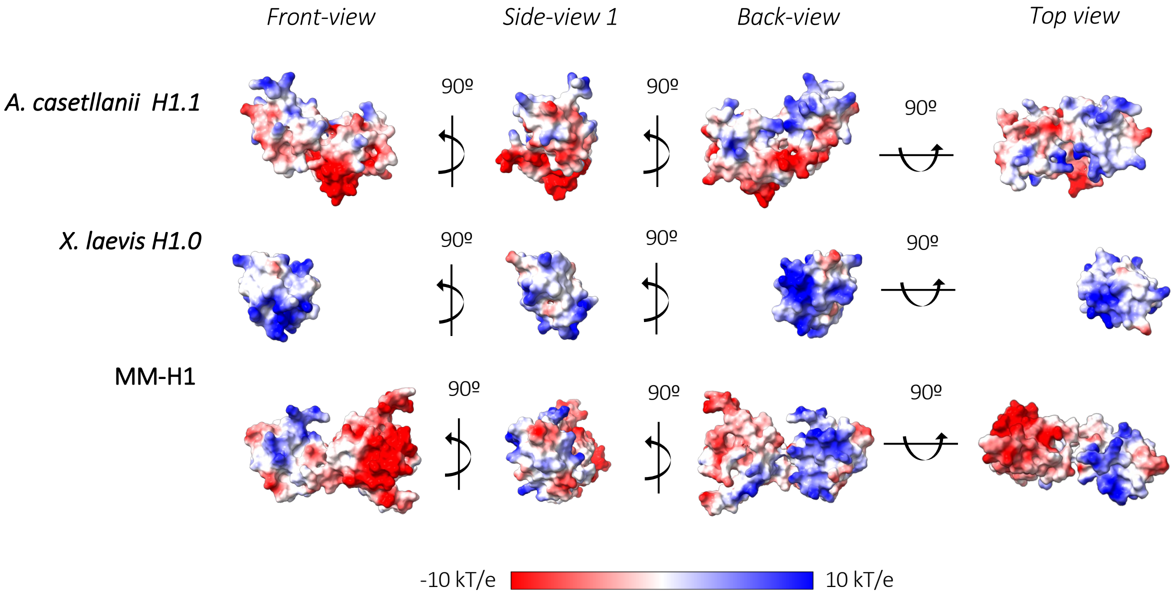

Supplementary Figure 6. Electrostatic surface representation comparison of host *A. castellanii* H1.1 and *X. laevis* H1.0 to *Medusa medusae* linker histone H1.

Charged surface representation of *A. castellanii* H1.1, *X. laevis* H1.0, and MM-H1 with rotational views. Coordinates for *X. laevis* H1 were acquired from 5NL0. Coordinates for *A. castellanii* H1.1 and MM-H1 were determined through AlphaFold. Related to Figure 6.

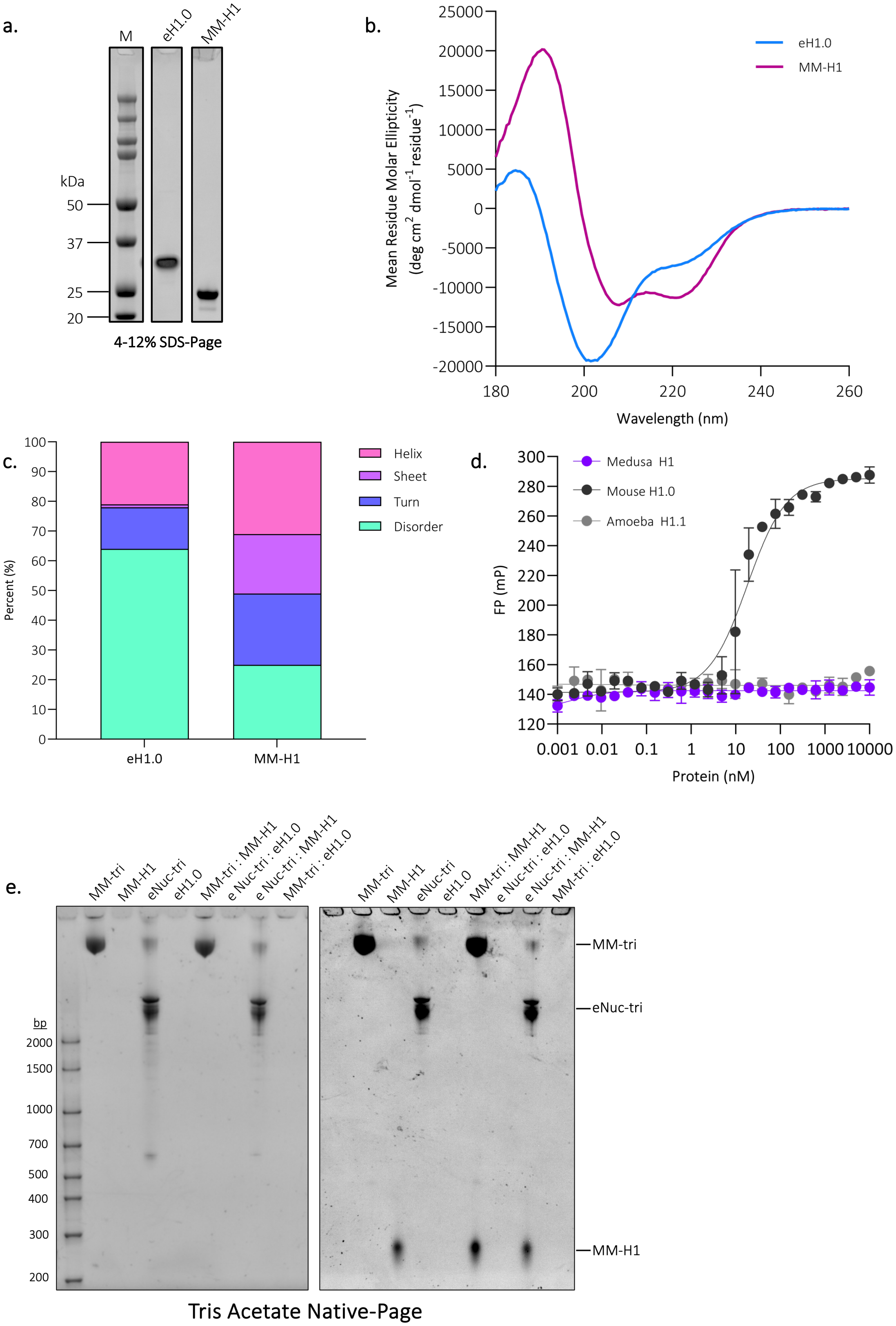

a.
M
eH1.0
MM-H1
kDa
50
37
25
20
4-12% SDS-Page
b.
Mean Residue Molar Ellipticity (deg cm^2 dmol^{-1} residue^{-1})
eH1.0
MM-H1
Wavelength (nm)
c.
Percent (%)
Helix
Sheet
Turn
Disorder
eH1.0
MM-H1
d.
FP (mP)
Medusa H1
Mouse H1.0
Amoeba H1.1
Protein (nM)
e.
MM-tri
MM-H1
eNuc-tri
eH1.0
MM-tri : MM-H1
e Nuc-tri : eH1.0
e Nuc-tri : MM-H1
MM-tri : eH1.0
bp
2000
1500
1000
700
500
400
300
200
MM-tri
eNuc-tri
MM-H1
Tris Acetate Native-Page

Supplementary Figure 7. Biochemical analysis of *Mus musculus* and *Medusavirus medusae* histone H1.

(a) Purified MM-putative linker histone H1 (MM-H1) and *Mus musculus* H1.0 (eH1.0). Experiment was repeated independently more than three times with similar results.

(b) CD spectra of purified eH1.0 (blue) and MM-H1 (pink). For each H1 protein, five replicate CD spectra were averaged, baseline-corrected for signal contributions by the buffer.

(c) Secondary structure estimation based on experimental CD data (shown in b), using DichroIDP.

(d) Fluorescence polarization of MM-H1, eH1.0 and Amoeba H1.1 with fluorescently labeled 25-mer DNA. Data points shown are the mean (SD as error bars, n=3).

(e) Gel shift of MM-H1 or eH1.0 with MM-tri or eNuc-Tri, analyzed on a Tris-Acetate gel (n=1), stained with EtBr (to visualized DNA, left) and Instant Protein Stain (to visualize protein, right).

Related to Figure 7. Source data are provided as a Source Data file.

Supplementary Table 1. Summary of cryoEM data collection and refinement.

	MM-NLP207 Crosslinked (EMDB- 42053) (PDB- 8UA7)	MM-NLP207 Native (EMDB-45981)
**Data collection and processing**		
Magnification	130,000	130,000
Voltage (kV)	300	300
Electron exposure (e–/Å^2)	50	46.29
Defocus range (μm)	0.6-1.7	0.8-2.2
Pixel size (Å)	0.97	1.017
Symmetry imposed	C1	C1
Initial particle images (no.)	2,270,549	545,861
Final particle images (no.)	159,734	72,786
Map resolution (Å)	3.3	4.9
FSC threshold	0.143	0.143
Map resolution range (Å)	2.8-6.0	4.5-8.5
**Refinement**		
Initial model used (PDB code)	1AOI	
Model resolution (Å) 0.143 FSC threshold	3.25	
Model resolution (Å) 0.5 FSC threshold	3.77	
Map versus model cross-correlation	0.77	
Model resolution range (Å)	2.8-6.0	
Map sharpening *B* factor (Å^2)	-51.7	
Model composition		
Non-hydrogen atoms	11265	
Protein residues	778	
Nucleotide	260	
Ligands	0	
B factors (min/max/mean)		
Protein	28.44/229.15/71.75	
Nucleotide	59.20/319.86/127.66	
R.m.s. deviations		
Bond lengths (Å)	0.004	
Bond angles (°)	0.676	
Validation		
MolProbity score	2.76	
Clashscore	12	
Poor rotamers (%)	5.54	
Ramachandran plot		
Favored (%)	88.98	
Allowed (%)	10.63	
Outliers (%)	0.39	
